# Methyl 2-[(*tert*-but­oxy­carbon­yl)amino]-3-(4-hy­droxy­phen­yl)propano­ate

**DOI:** 10.1107/S160053681301979X

**Published:** 2013-08-03

**Authors:** Xiaokun Li

**Affiliations:** a College of Pharmacy, Henan University of Traditional Chinese Medicine, Zhengzhou 450046, People’s Republic of China

## Abstract

In the title mol­ecule, C_15_H_21_NO_5_, the dihedral angle between the mean plane of the –N—C(=O)—O– group [maximum deviation = 0.002 (1) Å for the C atom] and the benzene ring is 82.2 (2)°. In the crystal, O—H⋯O and N—H⋯O hydrogen bonds connect the mol­ecules, forming a two-dimensional network parallel to (001).

## Related literature

For the biological activity of related compounds, see: Sykes *et al.* (1999 ▶).
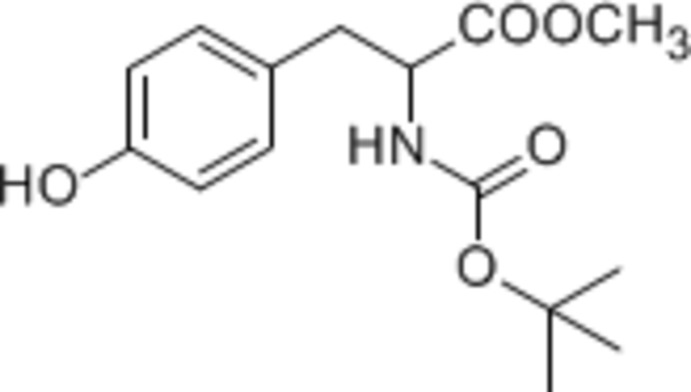


## Experimental

### 

#### Crystal data

C_15_H_21_NO_5_
*M* *_r_* = 295.33
Orthorhombic, 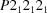

*a* = 8.7879 (8) Å
*b* = 9.4844 (9) Å
*c* = 18.9207 (18) Å
*V* = 1577.0 (3) Å^3^
*Z* = 4
Mo *K*α radiation
μ = 0.09 mm^−1^
*T* = 100 K
0.55 × 0.49 × 0.45 mm

#### Data collection

Bruker APEXII CCD diffractometer
Absorption correction: multi-scan (*SADABS*; Sheldrick, 1996 ▶) *T*_min_ = 0.962, *T*_max_ = 0.989
9339 measured reflections
3636 independent reflections
3469 reflections with *I* > 2σ(*I*)
*R*_int_ = 0.072

#### Refinement

*R*[*F* ^2^ > 2σ(*F* ^2^)] = 0.039
*wR*(*F* ^2^) = 0.102
*S* = 1.03
3636 reflections
190 parameters
H-atom parameters constrained
Δρ_max_ = 0.32 e Å^−3^
Δρ_min_ = −0.20 e Å^−3^

### 

Data collection: *APEX2* (Bruker, 2007 ▶); cell refinement: *APEX2*; data reduction: *SAINT* (Bruker, 2007 ▶)’; program(s) used to solve structure: *SHELXTL* (Sheldrick, 2008 ▶); program(s) used to refine structure: *SHELXTL*; molecular graphics: *SHELXTL* and *PLATON* (Spek, 2009 ▶); software used to prepare material for publication: *SHELXTL* and *publCIF* (Westrip, 2010 ▶).

## Supplementary Material

Crystal structure: contains datablock(s) I, global. DOI: 10.1107/S160053681301979X/lh5632sup1.cif

Structure factors: contains datablock(s) I. DOI: 10.1107/S160053681301979X/lh5632Isup2.hkl

Supplementary material file. DOI: 10.1107/S160053681301979X/lh5632Isup3.cml

Additional supplementary materials:  crystallographic information; 3D view; checkCIF report

## Figures and Tables

**Table 1:** Hydrogen-bond geometry (Å, °)

*D*—H⋯*A*	*D*—H	H⋯*A*	*D*⋯*A*	*D*—H⋯*A*
N1—H1*B*⋯O3^i^	0.86	2.27	3.0583 (16)	153
O1—H1*C*⋯O5^ii^	0.82	1.92	2.7356 (15)	180

Symmetry codes: (i) 
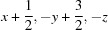
; (ii) 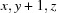.

**Fig. 1.**
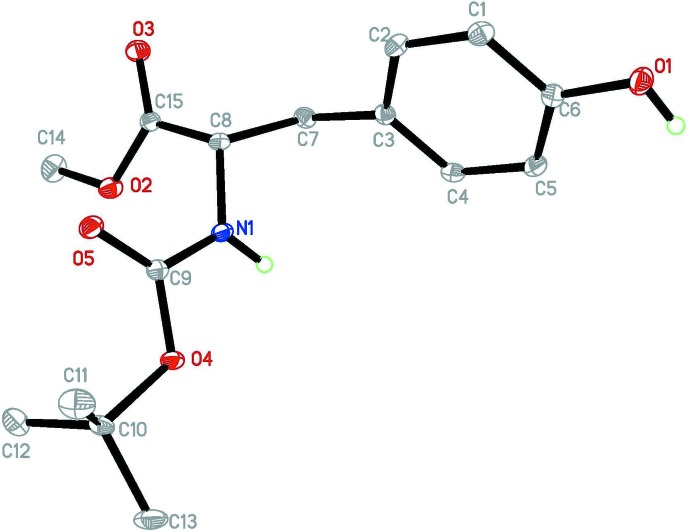
The molecular strcuture of the title compound with displacement ellipsoids drawn at the 30% probability level.

**Fig. 2.**
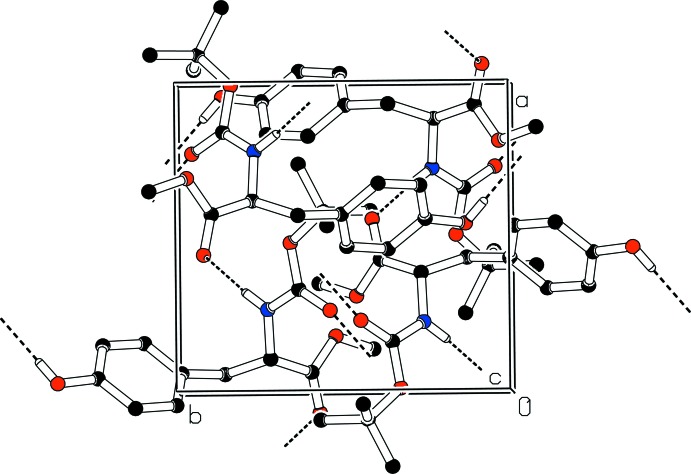
Part of the crystal structure with hydrogen bonds shown as dashed lines. H atoms bonded to C atoms are not shown.

## supplementary crystallographic information

### 1. Comment

Amide, ester and hydroxyl groups widely exist in many biologically active compounds or can be utilized in prodrugs (Sykes *et al.*, 1999). Herein we report the crystal structure of the title compound. The dihedral angle between the mean-plane of the amide group (N1/C9/O5/O4) [a maximum deviation of 0.002 (1)° for C9) and the benzene ring (C1–C6) is 82.2 (2)°. In the crystal, O—H···O and N—H···O hydrogen bonds connect molecules forming a two-dimensional network parallel to (001) (Fig. 2).

### 2. Experimental

The title compound (0.3 mmol, 88.5 mg) was dissolved in 10 ml of methanol solution. Colorless block-shaped crystals separated after 5 d.

### 3. Refinement

H atoms were included in calculated positions and treated as riding atoms: C—H = 0.93, 0.96 and 0.98 Å for CH(aromatic), CH_3_ and CH(methine) H atoms, respectively, or N—H = 0.86 Å and O—H = 0.82 Å with *U*_iso_(H)= *kU*_eq_(parent C-atom, N), where k = 1.5 for CH_3_ and hydroxyl H atoms and k = 1.2 for all other H atoms. The absolute congiuration could not be determined from the X-ray data. In the absence of anamolous dispersion effects Friedel pairs were merged.

### Crystal data

**Table:**

C_15_H_21_NO_5_	*F*(000) = 632
*M**_r_* = 295.33	*D*_x_ = 1.244 Mg m^−^^3^
Orthorhombic, *P*2_1_2_1_2_1_	Mo *K*α radiation, λ = 0.71073 Å
Hall symbol: P 2ac 2ab	Cell parameters from 9371 reflections
*a* = 8.7879 (8) Å	θ = 1.0–27.6°
*b* = 9.4844 (9) Å	µ = 0.09 mm^−^^1^
*c* = 18.9207 (18) Å	*T* = 100 K
*V* = 1577.0 (3) Å^3^	Block, colourless
*Z* = 4	0.55 × 0.49 × 0.45 mm

### Data collection

**Table:**

Bruker APEXII CCD diffractometer	3636 independent reflections
Radiation source: fine-focus sealed tube	3469 reflections with *I* > 2σ(*I*)
Graphite monochromator	*R*_int_ = 0.072
φ and ω scans	θ_max_ = 27.6°, θ_min_ = 2.2°
Absorption correction: multi-scan (*SADABS*; Sheldrick, 1996)	*h* = −11→9
*T*_min_ = 0.962, *T*_max_ = 0.989	*k* = −12→8
9339 measured reflections	*l* = −24→24

### Refinement

**Table:**

Refinement on *F*^2^	Primary atom site location: structure-invariant direct methods
Least-squares matrix: full	Secondary atom site location: difference Fourier map
*R*[*F*^2^ > 2σ(*F*^2^)] = 0.039	Hydrogen site location: inferred from neighbouring sites
*wR*(*F*^2^) = 0.102	H-atom parameters constrained
*S* = 1.03	*w* = 1/[σ^2^(*F*_o_^2^) + (0.0581*P*)^2^ + 0.1712*P*] where *P* = (*F*_o_^2^ + 2*F*_c_^2^)/3
3636 reflections	(Δ/σ)_max_ = 0.008
190 parameters	Δρ_max_ = 0.32 e Å^−^^3^
0 restraints	Δρ_min_ = −0.20 e Å^−^^3^

### Special details

**Table:**

Geometry. All e.s.d.'s (except the e.s.d. in the dihedral angle between two l.s. planes) are estimated using the full covariance matrix. The cell e.s.d.'s are taken into account individually in the estimation of e.s.d.'s in distances, angles and torsion angles; correlations between e.s.d.'s in cell parameters are only used when they are defined by crystal symmetry. An approximate (isotropic) treatment of cell e.s.d.'s is used for estimating e.s.d.'s involving l.s. planes.
Refinement. Refinement of *F*^2^ against ALL reflections. The weighted *R*-factor *wR* and goodness of fit *S* are based on *F*^2^, conventional *R*-factors *R* are based on *F*, with *F* set to zero for negative *F*^2^. The threshold expression of *F*^2^ > σ(*F*^2^) is used only for calculating *R*-factors(gt) *etc*. and is not relevant to the choice of reflections for refinement. *R*-factors based on *F*^2^ are statistically about twice as large as those based on *F*, and *R*-factors based on ALL data will be even larger.

### Fractional atomic coordinates and isotropic or equivalent isotropic displacement parameters (Å^2^)

**Table:**

	*x*	*y*	*z*	*U*_iso_*/*U*_eq_	
C1	−0.04196 (17)	1.13511 (15)	0.18071 (7)	0.0216 (3)	
H1A	−0.1123	1.1434	0.2172	0.026*	
C2	−0.03335 (16)	1.01206 (15)	0.14197 (7)	0.0205 (3)	
H2A	−0.0978	0.9376	0.1531	0.025*	
C3	0.07017 (15)	0.99675 (14)	0.08627 (7)	0.0172 (3)	
C4	0.16908 (15)	1.10800 (15)	0.07254 (7)	0.0192 (3)	
H4A	0.2409	1.0991	0.0367	0.023*	
C5	0.16250 (16)	1.23229 (15)	0.11142 (7)	0.0200 (3)	
H5A	0.2297	1.3055	0.1016	0.024*	
C6	0.05528 (16)	1.24689 (14)	0.16488 (7)	0.0184 (3)	
C7	0.06700 (17)	0.86702 (15)	0.04022 (7)	0.0188 (3)	
H7A	0.1336	0.8823	0.0001	0.023*	
H7B	−0.0354	0.8547	0.0221	0.023*	
C8	0.11575 (14)	0.73007 (14)	0.07772 (7)	0.0166 (3)	
H8A	0.0573	0.7217	0.1216	0.020*	
C9	0.33960 (15)	0.64306 (14)	0.13963 (7)	0.0159 (3)	
C10	0.59030 (15)	0.56854 (15)	0.18449 (7)	0.0199 (3)	
C11	0.5429 (2)	0.5555 (2)	0.26171 (8)	0.0302 (3)	
H11A	0.5434	0.6471	0.2833	0.045*	
H11B	0.6131	0.4950	0.2861	0.045*	
H11C	0.4424	0.5161	0.2644	0.045*	
C12	0.59178 (18)	0.42703 (17)	0.14678 (8)	0.0280 (3)	
H12A	0.6225	0.4403	0.0985	0.042*	
H12B	0.4917	0.3866	0.1480	0.042*	
H12C	0.6621	0.3649	0.1699	0.042*	
C13	0.74355 (17)	0.64103 (19)	0.17817 (9)	0.0305 (3)	
H13A	0.7714	0.6480	0.1292	0.046*	
H13B	0.8190	0.5872	0.2030	0.046*	
H13C	0.7373	0.7338	0.1982	0.046*	
C14	0.1469 (2)	0.42237 (17)	−0.04498 (8)	0.0292 (3)	
H14A	0.2370	0.3749	−0.0609	0.044*	
H14B	0.0943	0.4618	−0.0848	0.044*	
H14C	0.0816	0.3563	−0.0213	0.044*	
C15	0.07176 (16)	0.60783 (14)	0.02939 (7)	0.0180 (3)	
N1	0.27499 (13)	0.73700 (12)	0.09567 (6)	0.0170 (2)	
H1B	0.3301	0.8029	0.0778	0.020*	
O1	0.04029 (13)	1.36708 (11)	0.20398 (5)	0.0247 (2)	
H1C	0.1090	1.4220	0.1939	0.037*	
O2	0.18880 (11)	0.53485 (11)	0.00379 (5)	0.0221 (2)	
O3	−0.05943 (13)	0.58394 (11)	0.01458 (6)	0.0254 (2)	
O4	0.48955 (11)	0.66726 (10)	0.14566 (5)	0.0186 (2)	
O5	0.27010 (12)	0.54940 (10)	0.16994 (5)	0.0204 (2)	

### Atomic displacement parameters (Å^2^)

**Table:**

	*U*^11^	*U*^22^	*U*^33^	*U*^12^	*U*^13^	*U*^23^
C1	0.0215 (7)	0.0263 (7)	0.0171 (6)	−0.0023 (6)	0.0044 (5)	0.0007 (5)
C2	0.0205 (6)	0.0205 (6)	0.0206 (6)	−0.0054 (5)	0.0015 (5)	0.0015 (5)
C3	0.0161 (6)	0.0182 (6)	0.0172 (6)	0.0019 (5)	−0.0039 (5)	0.0011 (5)
C4	0.0145 (6)	0.0235 (7)	0.0195 (6)	0.0009 (5)	0.0020 (5)	0.0009 (5)
C5	0.0176 (6)	0.0208 (6)	0.0217 (6)	−0.0034 (5)	0.0006 (5)	0.0018 (5)
C6	0.0200 (6)	0.0185 (6)	0.0167 (5)	−0.0002 (5)	−0.0023 (5)	0.0006 (5)
C7	0.0179 (6)	0.0208 (6)	0.0178 (6)	0.0018 (5)	−0.0025 (5)	−0.0009 (5)
C8	0.0116 (6)	0.0203 (6)	0.0178 (6)	−0.0004 (5)	−0.0014 (5)	−0.0005 (5)
C9	0.0152 (6)	0.0171 (6)	0.0155 (5)	−0.0002 (5)	0.0007 (5)	−0.0038 (5)
C10	0.0145 (6)	0.0248 (7)	0.0206 (6)	0.0027 (5)	−0.0050 (5)	0.0017 (5)
C11	0.0281 (8)	0.0429 (9)	0.0196 (6)	0.0005 (7)	−0.0040 (6)	0.0012 (6)
C12	0.0281 (8)	0.0264 (7)	0.0296 (7)	0.0064 (6)	−0.0033 (6)	0.0000 (6)
C13	0.0146 (7)	0.0421 (9)	0.0348 (8)	−0.0032 (6)	−0.0069 (6)	0.0052 (7)
C14	0.0364 (9)	0.0281 (7)	0.0232 (7)	−0.0031 (7)	0.0026 (6)	−0.0084 (6)
C15	0.0164 (6)	0.0189 (6)	0.0188 (6)	−0.0003 (5)	−0.0011 (5)	0.0037 (5)
N1	0.0129 (5)	0.0185 (5)	0.0196 (5)	−0.0023 (4)	−0.0008 (4)	0.0012 (4)
O1	0.0269 (5)	0.0215 (5)	0.0256 (5)	−0.0057 (4)	0.0063 (4)	−0.0059 (4)
O2	0.0191 (5)	0.0249 (5)	0.0224 (5)	−0.0012 (4)	0.0020 (4)	−0.0060 (4)
O3	0.0179 (5)	0.0250 (5)	0.0333 (5)	−0.0016 (4)	−0.0080 (4)	−0.0020 (4)
O4	0.0123 (4)	0.0210 (5)	0.0223 (4)	−0.0010 (3)	−0.0038 (4)	0.0031 (4)
O5	0.0175 (5)	0.0214 (5)	0.0221 (4)	−0.0027 (4)	0.0013 (4)	0.0028 (4)

### Geometric parameters (Å, º)

**Table:**

C1—C2	1.380 (2)	C10—O4	1.4834 (16)
C1—C6	1.3942 (19)	C10—C13	1.517 (2)
C1—H1A	0.9300	C10—C12	1.520 (2)
C2—C3	1.3997 (19)	C10—C11	1.524 (2)
C2—H2A	0.9300	C11—H11A	0.9600
C3—C4	1.3915 (19)	C11—H11B	0.9600
C3—C7	1.5079 (18)	C11—H11C	0.9600
C4—C5	1.391 (2)	C12—H12A	0.9600
C4—H4A	0.9300	C12—H12B	0.9600
C5—C6	1.3893 (19)	C12—H12C	0.9600
C5—H5A	0.9300	C13—H13A	0.9600
C6—O1	1.3653 (16)	C13—H13B	0.9600
C7—C8	1.5408 (19)	C13—H13C	0.9600
C7—H7A	0.9700	C14—O2	1.4578 (17)
C7—H7B	0.9700	C14—H14A	0.9600
C8—N1	1.4415 (16)	C14—H14B	0.9600
C8—C15	1.5265 (18)	C14—H14C	0.9600
C8—H8A	0.9800	C15—O3	1.2079 (18)
C9—O5	1.2210 (17)	C15—O2	1.3309 (17)
C9—O4	1.3424 (16)	N1—H1B	0.8600
C9—N1	1.3447 (17)	O1—H1C	0.8200
			
C2—C1—C6	119.69 (13)	O4—C10—C11	111.29 (12)
C2—C1—H1A	120.2	C13—C10—C11	110.79 (12)
C6—C1—H1A	120.2	C12—C10—C11	112.37 (13)
C1—C2—C3	121.55 (13)	C10—C11—H11A	109.5
C1—C2—H2A	119.2	C10—C11—H11B	109.5
C3—C2—H2A	119.2	H11A—C11—H11B	109.5
C4—C3—C2	117.90 (12)	C10—C11—H11C	109.5
C4—C3—C7	121.49 (12)	H11A—C11—H11C	109.5
C2—C3—C7	120.51 (12)	H11B—C11—H11C	109.5
C5—C4—C3	121.20 (12)	C10—C12—H12A	109.5
C5—C4—H4A	119.4	C10—C12—H12B	109.5
C3—C4—H4A	119.4	H12A—C12—H12B	109.5
C6—C5—C4	119.85 (12)	C10—C12—H12C	109.5
C6—C5—H5A	120.1	H12A—C12—H12C	109.5
C4—C5—H5A	120.1	H12B—C12—H12C	109.5
O1—C6—C5	122.90 (12)	C10—C13—H13A	109.5
O1—C6—C1	117.34 (12)	C10—C13—H13B	109.5
C5—C6—C1	119.75 (12)	H13A—C13—H13B	109.5
C3—C7—C8	114.62 (10)	C10—C13—H13C	109.5
C3—C7—H7A	108.6	H13A—C13—H13C	109.5
C8—C7—H7A	108.6	H13B—C13—H13C	109.5
C3—C7—H7B	108.6	O2—C14—H14A	109.5
C8—C7—H7B	108.6	O2—C14—H14B	109.5
H7A—C7—H7B	107.6	H14A—C14—H14B	109.5
N1—C8—C15	114.93 (11)	O2—C14—H14C	109.5
N1—C8—C7	109.87 (11)	H14A—C14—H14C	109.5
C15—C8—C7	107.10 (10)	H14B—C14—H14C	109.5
N1—C8—H8A	108.2	O3—C15—O2	123.75 (13)
C15—C8—H8A	108.2	O3—C15—C8	121.54 (12)
C7—C8—H8A	108.2	O2—C15—C8	114.67 (11)
O5—C9—O4	125.14 (13)	C9—N1—C8	121.70 (11)
O5—C9—N1	124.15 (13)	C9—N1—H1B	119.2
O4—C9—N1	110.71 (12)	C8—N1—H1B	119.2
O4—C10—C13	101.82 (11)	C6—O1—H1C	109.5
O4—C10—C12	109.26 (11)	C15—O2—C14	114.55 (12)
C13—C10—C12	110.83 (13)	C9—O4—C10	121.33 (11)

### Hydrogen-bond geometry (Å, º)

**Table:**

*D*—H···*A*	*D*—H	H···*A*	*D*···*A*	*D*—H···*A*
N1—H1*B*···O3^i^	0.86	2.27	3.0583 (16)	153
O1—H1*C*···O5^ii^	0.82	1.92	2.7356 (15)	180

Symmetry codes: (i) *x*+1/2, −*y*+3/2, −*z*; (ii) *x*, *y*+1, *z*.
